# Supplementary material for: Interaction of clinical-stage antibodies with heme predicts their physiochemical and binding qualities
Source: Commun Biol. 2021 Mar 23;4:391. doi: 10.1038/s42003-021-01931-7 (PMC7988133; doi:10.1038/s42003-021-01931-7)
Supplement: Supplementary file 2 — Supplementary Information [file 42003_2021_1931_MOESM2_ESM.pdf]

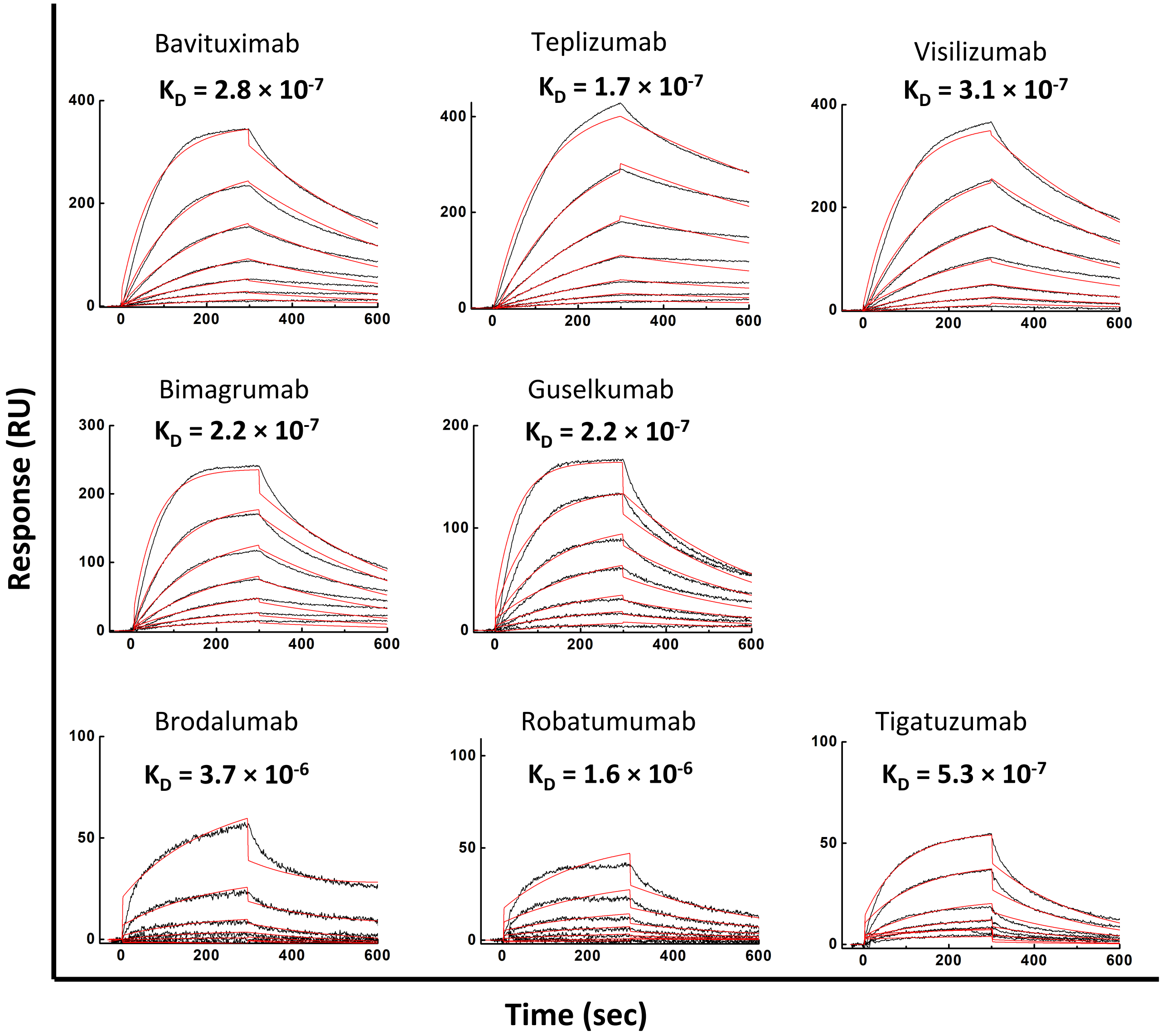

Supplementary Figure 1. Real time interaction profiles of binding of heme to immobilized Abs. The black line depicts the binding profiles obtained after injection of serial dilutions of hemin (1250 – 19.5 nM). The red lines depict the fits of data obtained by global analysis using Langmuir kinetic model. The fit of binding response for Brodalumab includes correction for drift of the baseline. The values of K_D indicated. All measurements were performed at 25 °C.
