# Supplementary material for: Interaction of clinical-stage antibodies with heme predicts their physiochemical and binding qualities
Source: Commun Biol. 2021 Mar 23;4:391. doi: 10.1038/s42003-021-01931-7 (PMC7988133; doi:10.1038/s42003-021-01931-7)
Supplement: Supplementary file 3 — Description of Additional Supplementary Files [file 42003_2021_1931_MOESM3_ESM.pdf]

Description of Additional Supplementary Files

File name: Supplementary Data 1

Description: Information about studied Abs.

File name: Supplementary Data 2

Description: Source data for Figures 1b, c; Figure 2b, c, d, and Figure 3a.
